# Operative Behandlung der Trigeminusneuralgie

**DOI:** 10.1007/s00482-024-00835-9

**Published:** 2024-10-17

**Authors:** Rezvan Ahmadi, Volker Martin Tronnier

**Affiliations:** 1 Neurochirurgische Klinik, Universitätsklinik Heidelberg, Im Neuenheimer Feld 400, 69120 Heidelberg, Deutschland; 2 Medizinische Fakultät, Universität zu Lübeck, Ratzeburger Allee 160, 23562 Lübeck, Deutschland

**Keywords:** Neuromodulation, Neuropathische Gesichtsschmerzen, Mikrovaskuläre Dekompression, Radiochirurgie, Thermokoagulation, Neuromodulation, Neuropathic facial pain, Microvascular decompression, Radiosurgery, Thermocoagulation

## Abstract

Die Schmerzchirurgie zur Behandlung von neuropathischen Schmerzen, zu denen auch die Trigeminusneuralgie (TGN) gehört, wird in die drei Gruppen der Dekompression, Ablation und Neuromodulation eingeteilt. Die Mikrovaskuläre Dekompression (MVD) ist die einzige kausale Therapie der TGN, die insbesondere im Falle der klassischen TGN auf Grund eines nachgewiesenen Gefäß-Nerven-Konflikts in Frage kommt. Zu den ablativen Verfahren gehören, neben den perkutanen, auch radiochirurgische Methoden, die vor allem bei der idiopathischen Trigeminusneuralgie zum Einsatz kommen. Bei irreversiblen Neuropathien des Nervus trigeminus gilt der Therapiealgorithmus anderer neuropathischer Schmerzen und gegebenenfalls der Einsatz neuromodulativer Verfahren. Für die Auswahl der Therapie müssen die Diagnose, die Nebenwirkungen der Medikamente und die individuellen Risiken der Patienten neben den Behandlungsergebnissen berücksichtigt werden (siehe aktuelle S1 Leitlinie der Deutschen Gesellschaft für Neurologie).

## Abstract (en)

Surgical procedures for treating neuropathic pain, including trigeminal neuralgia (TGN), are categorized into three groups: decompression, ablation, and neuromodulation. Microvascular decompression is the only causal therapy for TGN, applicable favorably in cases of classical TGN due to a vascular nerve conflict. Ablative procedures include both percutaneous and radiosurgical methods, which are mainly used for idiopathic trigeminal neuralgia. For irreversible neuropathies of the trigeminal nerve, the algorithm for other neuropathic pain conditions should be considered, along with the potential use of neuromodulatory techniques. When selecting a therapy, diagnosis, medication side effects, individual patient risks, and treatment outcomes must all be taken into account (current S1 guideline from the German Society of Neurology).

## Lernziele

Nach Lektüre dieses Beitrags

- können Sie die Methoden der kranialen Schmerzchirurgie zur Behandlung von Trigeminusneuralgien (TGN) benennen.
- kennen Sie die relevante Einteilung der TGN unter Berücksichtigung der pathophysiologischen Grundlagen.
- können Sie die operativen Indikationen und Ergebnisse benennen,
- kennen Sie die Risiken und Limitationen der verschiedenen Verfahren.
- können Sie Kriterien zur Auswahl der Methoden und Aufklärung der Patienten einsetzen.
- Sind Ihnen die empfohlenen Qualifikationen für die chirurgischen Behandlungen der TGN bekannt.

## Einleitung

Die klassische Trigeminusneuralgie ist als blitzartig einschießender, heftiger, elektrisierender und stechender Schmerz im Versorgungsgebiet eines oder mehrerer Trigeminusäste definiert [1, 2]. Die Attacken halten typischerweise Sekunden an und treten sowohl spontan als auch durch nichtschmerzhafte Reize (sogenannte Trigger) wie Berührung im Trigeminusareal, Kauen, Sprechen, Schlucken oder Zähneputzen auf. Die Schmerzen sind invalidisierend und quälend und lassen oft eine Nahrungsaufnahme nicht mehr zu. Daher muss jede Trigeminusneuralgie umgehend medikamentös behandelt werden [3]. Die Lebenszeitprävalenz ist 0,16–0,3 % [4].

### Fallbeispiel

**Abb. 1:**
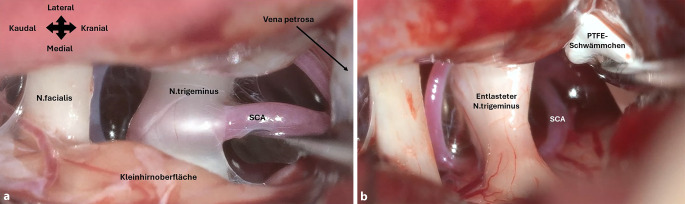
Intraoperative Befunde bei unserem Fallbeispiel. **a** Kompression und Eindellung des re. Nervus trigeminus durch eine Arteria-cerebelli-superior(SCA)-Schlinge bei einem 53-jährigen Patienten. **b** Verlagerung und Fixierung mittel Polytetrafluorethylen(PTFE)-Schwämmchen und Fibrinkleber zur Dekompression

Ein 53-jähriger Patient wird in einer für kraniale Schmerzchirurgie spezialisierten neurochirurgischen Klinik vorstellig. Er habe vor fünf Jahren erstmalig attackenartige Schmerzen im rechten Unterkiefer erlitten. Die Schmerzen seien sehr stark, elektrisierend und blitzartig und immer entlang der unteren Zahnreihe rechts bis zur Mitte. Anfänglich hätten Schmerzmedikamente gut geholfen. Seit vier Monaten habe er zunehmende Schmerzen, weshalb er zusätzliche Medikamente einnehme (Carbamazepin, Pregabalin, Amitriptylin). Darunter sei er einigermaßen schmerzfrei, müsse allerdings wegen der medikamentösen Nebenwirkungen überwiegend im Bett liegen. Attacken können durch Luftzug, Berührung, kalte Getränke oder Kauen ausgelöst werden. Er habe deshalb seit Wochen nichts Festes mehr gegessen und habe 10 kg abgenommen. Das cMRT zeigt neben einem Gefäß-Nerven-Kontakt im rechten Kleinhirnbrückenwinkel keine weiteren Auffälligkeiten. Nach der MVD (Abb. 1a, b) ist der Patient inzwischen seit drei Jahren schmerzfrei und benötigt keine Medikation.

Die **klassische Trigeminusneuralgie**

klassische Trigeminusneuralgie

ist durch eine vaskuläre Kompression des N. trigeminus am Abgang aus der Brücke bedingt. Am häufigsten durch die Arteria cerebelli superior, seltener durch die Arteria cerebelli anterior inferior, die Arteria basilaris oder hirnstammnahe Venen [5, 6]. Für die Ausprägung des „neurovaskulären Konflikts“ sind semiquantitative Einteilungen beschrieben [7, 8]. Ein im MRT nachgewiesener bloßer Gefäß-Nerven-Kontakt muss nicht zwangsläufig mit einer Trigeminusneuralgie einhergehen. So sind gelegentlich bilaterale Konflikte bei unilateraler TGN beschrieben; ebenso müssen sekundäre Neuralgien durch andere Veränderungen im Schmerzleitungssystem (wie z. B. bei einer Multiple Sklerose oder anderen Degenerationen) bedacht werden [9].

Bei eindeutiger klinischer Symptomatik ohne Nachweis eines neurovaskulären Konflikts auf hochauflösenden T2-gewichteten MR-Sequenzen und/oder TOF-MR-Angiographie spricht man von einer **idiopathischen Trigeminusneuralgie**

idiopathischen Trigeminusneuralgie

[10, 11, 12].

Da die **Trigeminusneuropathie** völlig anderen therapeutischen – auch chirurgischen – Prinzipien unterliegt, gelten die folgenden Empfehlungen für die chirurgischen Verfahren 1–3 für die klassische, die idiopathische und sekundäre Neuralgie (Tab. 1).

**Tab. 1:** Klassifikation der Trigeminusneuralgie und Differenzialdiagnose schmerzhafte Trigeminusneuropathie**. **(Aus der S1 Leitlinie der Deutschen Gesellschaft für Neurologie [13])

Klassische Trigeminusneuralgie	Sekundäre Trigeminusneuralgie	Idiopathische Trigeminusneuralgie	Schmerzhafte Trigeminusneuropathie
Wiederkehrende paroxysmale einseitige Gesichtsschmerzattacken, die die Kriterien für eine Trigeminusneuralgie erfüllen			Gesichtsschmerz im/in den Versorgungsbereich(en) eines oder mehrerer Äste des N. trigeminus, der durch eine andere Erkrankung verursacht wird
Paroxysmal mit oder ohne anhaltenden Dauerschmerz			Üblicherweise Dauerschmerz oder annähernder Dauerschmerz, kurze paroxysmale Schmerzattacken können auftreten, jedoch nicht vorherrschend
Neurovaskuläre Kompression mit morphologischen Veränderungen der Wurzel des Trigeminusnervs	Nachgewiesene Grunderkrankung, die die Neuralgie verursachen kann (MRT)	Keine Auffälligkeiten im MRT (Ausschluss klassische oder sekundäre Trigeminusneuralgie)	Hinweis auf neuronale Schädigung, neuropathische Schmerzen (brennender oder nadelstichartiger Dauerschmerz, sensible Defizite, Hyperalgesie/Allodynie)

## Chirurgische Methoden

Nach Ausschöpfung medikamentöser Therapien [3] kommen die folgenden (radio-)chirurgischen Maßnahmen in Betracht

1. **Mikrovaskuläre parapontine Dekompression**
  Mikrovaskuläre parapontine Dekompression
  (sogenannte Operation nach Jannetta)
2. Perkutane Verfahren
  Selektive Thermokoagulation
  Retroganglionäre Glycerolinstillation
  Ballonkompression
3. Radiochirurgie
4. Neuromodulation (TKMS, tDCS oder invasive PNS, sTNFS, THS und MCS)

### Cave

Medikamentöse Therapieresistenz oder intolerable Nebenwirkungen sind Gründe für die Überprüfung chirurgischer Behandlungsmöglichkeiten der TGN.

### Mikrovaskuläre Dekompression (Operation nach Jannetta)

#### Historie.

Die Dekompression des Nervus trigeminus am Kleinhirnbrückenwinkel geht zurück auf die 1920er und 1930er-Jahre, als die komplette oder partielle Durchtrennung (Rhizotomie) über eine retrosigmoidale Kraniotomie die Routineoperation darstellte. Dandy beschrieb 1934 215 Fälle mit einer Kompression des N. trigeminus durch die obere Kleinhirnarterie (SCA) in 30,7 % sowie einer venösen Kompression in weiteren 14 %. Er konnte bereits einen Zusammenhang zwischen der Kompression des proximalen Abschnitts des Nervs und den attackenartigen Schmerzen (Tic douloureux) herstellen [14]. Obwohl diese Beobachtung und deren Relevanz für die Therapie der TGN zunächst unterschätzt und teilweise angezweifelt wurden, starteten die ersten Versuche der dekompressiven Operationen, die teilweise noch eine ablative Komponente beinhalteten. Gardner zeigte die ersten Ergebnisse der Dekompression durch Interposition mit gelartigen Pads zwischen N. trigeminus und der komprimierenden Arterie über einen subokzipitalen Zugang und ohne zusätzliche Manipulation am Nervus trigeminus bei sechs Patienten [15].

Peter Joseph Jannetta entwickelte dann die Theorie der Kompression der Hirnnerven durch Gefäße, den sog. **Gefäß-Nerven-Konflikt**

Gefäß-Nerven-Konflikt,

als Ursache der Trigeminusneuralgie bzw. anderer Funktionsstörungen wie beispielsweise des Hemispasmus facialis weiter. Er führte 1966 die MVD mikrochirurgisch erstmalig bei TGN durch und fand die SCA als Ursache der Kompression, die durch Verlagerung und Interposition mit Schwämmchen aus hydroxyliertem Polyvinylacetal beseitigt werden konnte [16].

Inzwischen stellt die MVD als einzige kausale Therapie der klassischen TGN bei **medikamentöser Therapieresistenz**

medikamentöser Therapieresistenz

oder intolerablen Nebenwirkungen die Methode der Wahl dar. Hierbei muss eine individuelle Entscheidung anhand der chirurgischen und nichtchirurgischen Risiken erfolgen.

#### Merke

Bei therapierefraktärer klassischer Trigeminusneuralgie sollte immer die spezifische Diagnostik auf das Vorliegen eines neurovaskulären Konflikts eingeleitet und ggf. die neurochirurgische Beratung zur Behandlung mittels mikrovaskulärer Dekompression angeboten werden.

#### *Operative Technik*.

Der **retrosigmoidale Zugang**

retrosigmoidale Zugang

ist die gängigste Methode zum Erreichen des Kleinhirnbrückenwinkels und wird für die MVD bei TGN empfohlen. SCA (70–80 %) und die Arteria cerebelli anterior inferior (AICA,10–15 %) sind die häufigsten arteriellen Ursachen eines Gefäß-Nerven-Konflikts. Venen kommen weniger häufig (ca. 10 %) vor [17]. Die Nutzung von Teflon- oder Polytetrafluorethylen-Interponaten ist am weitesten verbreitet. Es wird diskutiert, ob die reine Verlagerung des Gefäßes (**Transposition**

Transposition)

eine nachhaltigere Wirkung ergibt, da auf Dauer keine potenzielle Kompression durch das Interponat selbst entsteht [18, 19, 20].

#### *Outcome*.

Eine Metaanalyse von neun aktuellen Studien, die eine hohe Qualität aufwiesen, bestätigt, dass die MVD zu einer initialen Schmerzfreiheit ohne Begleitmedikation (Barrow Neurological Institute Pain Intensity Scores BN I) in 82,9 % der Fälle führt (95 % CI 74,9–88,9 %, *p* < 0,01, I^2^ = 93,4 %), während lediglich 2,6 % als chirurgische Versager (BN IV–V) betrachtet werden können. Dieser schmerzfreie Effekt wird für 64,7 % der Patienten bei der letzten Nachkontrolle (nach 3–10 Jahren) berichtet [17]. Auch der Vergleich der beiden operativen Techniken in kurzfristigem postoperativem Ergebnis zeigt eine Schmerzfreiheit bei lediglich dekomprimierten Patienten mit Transposition ohne Interponat von 88,3 % versus 75 % bei Patienten mit Dekompression mit Interponat [17].

*Die häufigsten Komplikationen* dieser Operation sind eine Hörminderung oder Hörverlust (1,1 %) [17]. Außerdem sind temporäre und permanente Hypästhesien der betroffenen Gesichtsseite in bis zu 2,1 % bzw. 0,1 % berichtet worden [17]. Zur postoperativen Liquorleckage werden unterschiedliche Werte von 0,2 bis 8,2 % gefunden, die abhängig von der Methode des Duraverschlusses bzw. der Duraplastik nicht ausgewertet werden können [17]. Wichtig ist, dass kein Unterschied zwischen älteren, > 65-jährigen und jüngeren Patienten in Bezug auf Komplikationsraten gesehen werden konnte [21].

#### Merke

Hohes Alter per se ist keine Kontraindikation für chirurgische Eingriffe.

### Perkutane Verfahren am Ganglion Gasseri

Im Gegensatz zur mikrovaskulären Dekompression des N. trigeminus sind alle perkutanen Methoden destruierende (läsionelle) Verfahren, bei denen der N. trigeminus im Ganglion Gasseri thermisch (Thermokoagulation), chemisch (Glycerinrhizolyse) oder mechanisch (Ballonkompression) geschädigt wird. **Perkutane Verfahren**

Perkutane Verfahren

sind indiziert bei der **klassischen Trigeminusneuralgie**, wenn

- der Patient keine MVD wünscht,
- das Operationsrisiko *oder* Narkoserisiko für den Patienten zu hoch ist,

bei der **idiopathischen **Trigeminusneuralgie,

- da kein Gefäßnervenkonflikt vorhanden ist

und bei der **sekundären Neuralgie**

sekundären Neuralgie,

wenn

- die schmerzerzeugende Ursache nicht oder nur teilweise beseitigt werden kann (z. B. Tumoren des Kleinhirnbrückenwinkels),
- eine durch Multiple Sklerose bedingte Neuralgie vorliegt.

Hierbei müssen die perkutanen Verfahren mit der ebenfalls neuroläsionellen Radiochirurgie abgewogen werden. Vorteile der Radiochirurgie im Vergleich zu den perkutanen Verfahren ist die Nichtinvasivität, Nachteile sind der spätere Wirkungseintritt und die geringere Erfolgsrate im Langzeitverlauf.

Es wird immer wieder diskutiert, ob bei der idiopathischen Neuralgie nicht doch eine mikrovaskuläre Dekompression durchgeführt werden sollte, weil man einen neurovaskulären Konflikt im MRT übersehen haben könnte. Hierzu ist anzumerken, dass mit den aktuellen MR-Sequenzen eine Sensitivität von über 85 % und eine Spezifizität von 100 % für den Nachweis eines neurovaskulären Konflikts erbracht werden kann. Man konnte bereits zeigen, dass **automatische Sequenzierungen**

automatische Sequenzierungen

und **Multi-imaging Fusion**

Multi-imaging Fusion

die Sensitivität erhöhen ([22, 23]; Tab. 2).

**Tab. 2:** Empfohlene MR-Sequenzen zur lokalen Beurteilung des N. trigeminus bei (nicht sekundärer) Trigeminusneuralgie. (Mod. nach S1 Leitlinie der Deutschen Gesellschaft für Neurologie [13])

Allgemeine Beschreibung	Auflösung	Indikation
Stark T2-gewichtete Sequenz mit hoher, isotroper Ortsauflösung. Basierend entweder auf balanced Steady-State Free Precession(bSSFP)- oder 3D-Turbo-Spin-Echo(3D-TSE)-Technik	Isotrope Auflösung Voxelgröße < 1 mm^3^	Anatomische Beurteilung des zisternalen Verlaufs des N. trigeminus, Lagebeziehung insbesondere zu vaskulären Strukturen
		Detektion von Hämorrhagien, Hämosiderin
Kontrastmittelfreie, hochaufgelöste Darstellung der intrakraniellen Arterien (TOF-MRA)	0,8–1 mm isotrop	Differenzierung zwischen arteriellen und venösen Gefäßstrukturen. Ggf. Fusion mit T2-gewichteter Sequenz
Hochaufgelöste, kontrastmittelgestützte, T1-gewichtete Sequenz (idealerweise auch nativ akquiriert)	1 mm isotrop	Detektion einer Kontrastmittelaufnahme an einem möglichen Gefäßnervenkontakt. Präoperative Planung

Bei der **Thermokoagulation**

Thermokoagulation

oder** temperaturgesteuerten Radiofrequenzläsion**

temperatur-gesteuerten Radiofrequenzläsion

wird der N. trigeminus im Ganglion Gasseri nach Teststimulation **selektiv thermisch geschädigt**

Selektive thermische Schädigung

[24]. Das heißt, es werden durch die Höhe der Temperatur bedingt vor allem die dünnen, nicht myelinisierten schmerz- und temperaturleitenden Aδ- und C‑Fasern ausgeschaltet. Vor der Läsion wird eine Teststimulation durchgeführt, die es erlaubt. selektiv die einzelnen Nervenäste zu erreichen bzw. den ersten Trigeminusast zu schonen (typische Läsionsparameter sind 72–75 C° über 75–120 s). Wenn die Koagulationsnadel in den Röntgenaufnahmen korrekt im Foramen ovale positioniert ist, wird der Mandrin entfernt. In zwei Dritteln der Fälle treten einige Tropfen Liquor aus. Der Mandrin wird durch eine Thermosonde ersetzt, die die Messung der Temperatur während der Koagulation erlaubt. Neben der Höhe und der Dauer der Temperatur sind die Nadeldicke und die Länge der unisolierten Spitze für die Läsionsgröße entscheidend.

Kanpolat beschreibt in der größten retrospektiven Kohorte eine initiale Schmerzfreiheit von knapp 98 % und anhaltend gute Ergebnisse nach 5, 10, 15 und 20 Jahren in 58 %, 52 %, 42 und 41 % der Fälle [25]. Dies wird auch von Orhurhu et al. [26] bestätigt. Die Autoren zeigen in einer Metaanalyse zur Anwendung von RF für die Therapie von Gesichtsschmerzen, dass diese Behandlung eine hohe Effektivität sowohl in der sofortigen Kontrolle der Schmerzen (bis zu 100 %) als auch in der Langzeitbeobachtung (Schmerzfreiheit 83,3–92,3 % nach zwei Jahren) aufweist. Erwünscht ist eine Hypalgesie und Thermhypästhesie für den betroffenen Ast. Verminderungen der Berührungsempfindlichkeit (beim dritten Ast auch an der Zunge und Wangenschleimhaut) sind meist vorübergehend. Gelegentlich entstehen bei der Punktion ein Hämatom der Wange und eine temporäre Kieferöffnungsschwierigkeit. Der erste Ast sollte nicht lädiert werden, um eine Keratitis paralytica zu vermeiden. Schwerwiegende Komplikationen wie Doppelbilder, Meningitis, eine An- oder Dysaesthesia dolorosa sind sehr selten (unter 1 %). Mortalität wurde in der Metaanalyse nicht berichtet.

Aufgrund der spezifischeren Wirkung hat sich die **Thermokoagulation**

Thermokoagulation

gegenüber Glycerinrhizolyse und Ballonkompression durchgesetzt. Dies wird auch durch eine Metaanalyse gestützt, die diese drei perkutanen Verfahren hinsichtlich Langzeiteffektivität und Komplikation verglich [27]. Die perkutanen Eingriffe können im Falle eines Rezidivs mehrfach wiederholt werden. Im Allgemeinen nimmt jedoch das sensible Defizit nach multiplen Interventionen zu. Die Erfolgsaussichten sind nach einem wiederholten Eingriff häufig weniger gut als beim Ersteingriff.

#### Cave

Die perkutane Punktion und Thermokoagulation des Ganglion Gasseri stellt die einzige verfügbare Methode zur selektiven Ablation einzelner Äste des N. trigeminus dar.

### Radiochirurgische Behandlung

Für Patienten, die keine invasiven Verfahren wünschen oder aus medizinischen Gründen tolerieren, bietet sich die **Radiochirurgie**

Radiochirurgie

als **läsionelles Verfahren**

läsionelles Verfahren

an. Hierbei wird in der Regel der Trigeminushauptstamm parapontin im Bereich der Wurzeleintrittszone als Zielpunkt gewählt. Der entscheidende Unterschied zur mikrovaskulären Dekompression oder den perkutanen Verfahren ist der **verzögerte Wirkungseintritt**

verzögerte Wirkungseintritt

(in der Regel nach 2–4 Wochen) [28]. Typischerweise werden 80–90 Gy in einer Sitzung verabreicht. Der schmerzlindernde Effekt und das Risiko von Nebenwirkungen (in erster Linie Hyp- und Dysästhesien) steigt mit steigender Bestrahlungsdosis [29]. Im Vergleich zu den perkutanen Verfahren ist die Anzahl initial schmerzfreier Patienten geringer, die Langzeitergebnisse für die radiochirurgisch behandelten Patienten schlechter; Schmerzrezidive treten ebenfalls nach 1–2 Jahren auf, wobei eine Zweit- oder Drittbestrahlung erneut erfolgreich sein kann [30, 31].

### Neuromodulation

Besteht eine **irreversible Schädigung**

irreversible Schädigung

im sensomotorischen System, z. B. durch Traumata (zygomatico-maxilläre Frakturen), dentale oder kieferchirurgische Eingriffe, vorausgegangene neurochirurgische neuroläsionelle Verfahren, Infektionen, z. B. eine postzosterische Neuralgie– gekennzeichnet durch vorherrschende Dauerschmerzen, häufig brennenden Charakters, Schwellungsgefühle und ein sensomotorisches Defizit –, spricht man von einer **Trigeminusneuropathie**

Trigeminusneuropathie

**.** In diesen Fällen ist die mikrovaskuläre Dekompression nicht hilfreich (selbst bei nachgewiesenem Gefäßnervenkonflikt) und es sind weitere destruktive bzw. neuroläsionelle perkutane oder radiochirurgische Verfahren kontraindiziert. Vielmehr sollten hier** neuromodulative nichtinvasive**

neuromodulative nicht-invasive Verfahren

(TKMS, tDCS) oder **invasive Verfahren**

invasive Verfahren

(PNS, sTNFS, DBS und MCS) zum Einsatz kommen, die momentan im Sinne von individuellen Heilversuchen indiziert und an spezialisierten Zentren für kraniale Schmerzchirurgie eingesetzt werden können.

#### Merke

Neuromodulative Verfahren sind nicht- oder minimal-invasive Methoden zur effektiven Linderung von irreversiblen neuropathischen Schmerzen auch im Versorgungsgebiet des N. trigeminus.

## Fazit für die Praxis

- Die Schmerzchirurgie kann zur Therapie der Trigeminusneuralgie (TGN) eingesetzt werden. Die mikrovaskuläre Dekompression (MVD) stellt die einzige kausale Therapie der TGN dar und wird insbesondere bei klassischer TGN angewendet, wenn ein Gefäß-Nerven-Konflikt oder andere einengende Pathologien des N. trigeminus vorliegen. Zu den ablativen Verfahren gehören neben den perkutanen auch die radiochirurgischen Methoden, die vor allem bei der idiopathischen Trigeminusneuralgie und sekundären Formen zum Einsatz kommen und eine symptomatische Behandlung darstellen.
- Bei irreversiblen Neuropathien des Nervus trigeminus gilt der Therapiealgorithmus anderer neuropathischer Schmerzen und gegebenenfalls der Einsatz neuromodulativer Verfahren. Für die Auswahl der Therapie müssen die spezifische Gesichtsschmerzdiagnose, die medikamentösen Nebenwirkungen und die individuellen Patientenrisiken berücksichtigt werden. Das behandelnde Zentrum sollte Erfahrungen mit den medikamentösen und mehreren operativen Therapieverfahren haben, um anhand der Behandlungsergebnisse die für den individuellen Patienten geeignete Therapie empfehlen zu können.
- Für die umfassende Beurteilung, Beratung und Durchführung der operativen Therapien sind gründliche Kenntnisse und Handlungsexpertise der Neurochirurgen vorauszusetzen. Seit 2021 können neurochirurgische Fachärzte die o. g. Expertise und die Personenzertifizierung der Neurochirurgischen Akademie (NCA) unter der Schirmherrschaft der beiden neurochirurgischen Fachgesellschaften Deutsche Gesellschaft für Neurochirurgie (DGNC) und Berufsverband Deutsche Neurochirurgie (BDNC) erwerben.

## CME-Fragebogen

Eine 76-jährige Patientin berichtet über seit zwei Wochen blitzartig einschießende, heftige, elektrisierende Schmerzen im Versorgungsgebiet des 3. Trigeminusastes rechts. Die Attacken halten typischerweise Sekunden an und treten sowohl spontan als auch durch nichtschmerzhafte Reize wie Berührung im Trigeminusareal, Kauen, Sprechen, Schlucken oder Zähneputzen getriggert auf. Welche Diagnose ist am wahrscheinlichsten?

Klassische Trigeminusneuralgie

Sekundäre Trigeminusneuralgie

Idiopathische Trigeminusneuralgie

Schmerzhafte Trigeminusneuropathie

Zahnschmerzen bei Karies

Was ist die Schmerzursache bei der klassischen Trigeminusneuralgie?

Entmarkungen durch Multiple Sklerose

Neurovaskulärer Konflikt

Blutung im Gebiet der Hirnstammkerne des N. trigeminus

Somatoforme Schmerzstörung

Zahnhalskaries

Mit welcher Soforttherapie sollte bei Diagnose einer klassischen Trigeminusneuralgie begonnen werden?

Mikrovaskuläre Dekompressions-OP nach Jannetta

Medikamentöse Behandlung z. B. mit Carbamazepin

Selektive Thermokoagulation

Radiochirurgie

Invasive Neuromodulation (z. B. Tiefe Hirnstimulation)

Welches der folgenden Gefäße ist bei einem neurovaskulären Konflikt als Ursache der klassischen Trigeminusneuralgie am häufigsten involviert?

Arteria cerebelli superior

Arteria cerebelli anterior inferior

Arteria basilaris

Hirnstammnahe Venen

Arteria carotis interna

Was ist der Standardzugang für die Durchführung der mikrovaskulären Dekompression zur Behandlung der klassischen Trigeminusneuralgie?

Subtemporal

Transtentoriell

Retrosigmoidal

Subokzipital-Mittellinie

Transzerebellär

Welche der folgenden Symptome/Befunde stellen eine Kontraindikation für die mikrovaskuläre Dekompression dar?

Irreversible Neuropathie mit Dauerschmerzen und Hypästhesie im betroffenem Versorgungsareal

Hohes Patientenalter

Bildgebender Hinweis auf eine venöse Kompression

Patientenwunsch

Schmerzen in den Versorgungsgebiete von mehr als einem Ast des N. trigeminus

Eine 40-jährige Patientin berichtet seit einiger Zeit über einen brennenden Dauerschmerz im 2. Trigeminusast links mit zusätzlichen kurzen paroxysmalen Schmerzattacken, die jedoch nicht im Vordergrund stehen. Es besteht eine Hypästhesie und Allodynie im Bereich der linken Wange. Welche Diagnose ist am wahrscheinlichsten?

Klassische Trigeminusneuralgie

Sekundäre Trigeminusneuralgie

Idiopathische Trigeminusneuralgie

Schmerzhafte Trigeminusneuropathie

Gesichtsschmerz durch intrakraniellen Gefäß-Nerven-Kontakt

Eine 67-jährige Patientin mit einer Vorgeschichte der Multiplen Sklerose leidet seit sieben Jahren unter attackenartigen Schmerzen im linken Ober- und Unterkiefer. Die Sensibilität und Motorik im Gesicht sind intakt. Die medikamentöse Therapie ist ausgeschöpft und der Schmerz unkontrolliert. Das kraniale Magnetresonanztomogramm (cMRT) kann einen neurovaskulären Konflikt nicht ausschließen. Welche der folgenden Methoden sollten Sie ihr als Methode der Wahl vorschlagen?

Motor-Cortex-Stimulation (MCS)

Transkranielle Magnetstimulation (TKMS)

Mikrovaskuläre Dekompression

Perkutane Thermokoagulation des linken Ganglion Gasseri

Radiochirurgie des N. trigeminus-Kerngebiets

Welche Aussage zu perkutanen läsionellen Verfahren zur Behandlung einer Trigeminusneuralgie trifft zu?

Die initiale Ansprechrate ist insbesondere bei der Thermokoagulation des N. trigeminus im Ganglion Gasseri noch gering.

Die perkutanen Verfahren erreichen in der Regel keine langfristige Schmerzfreiheit.

Neben der erwünschten Hypalgesie und Thermhypästhesie für den betroffenen Ast können perkutane läsionelle Verfahren zu einer Hypästhesie im selben Areal als Nebenwirkung führen.

Perkutane läsionelle Verfahren werden an allen 3 Trigeminusästen gleichermaßen durchgeführt.

Aufgrund der spezifischeren Wirkung auf un- oder dünnmyelinisierte Schmerzfasern hat sich die Ballonkompression bei der Behandlung der klassischen Trigeminusneuralgie gegenüber den anderen nicht selektiven läsionellen Verfahren durchgesetzt.

Welche der folgenden Methoden der Neuromodulation stellt eine nicht-invasive Behandlung der klassischen Trigeminusneuralgie dar?

Neurostimulation des Ganglion Gasseri

Subkutane Trigeminus-Nerven- und Feldstimulation

Tiefe Hirnstimulation

Motor Cortex Stimulation

Transkranielle Magnetstimulation

## Abkürzungen

AICA: Arteria cerebelli inferior anterior
BDNC: Berufsverband Deutsche Neurochirurgie
bFFE: Balanced fast field echo
cMRT: Kraniales Magnetresonanztomogramm
DBS: Deep brain stimulation
DGNC: Deutsche Gesellschaft für Neurochirurgie
Gy: Gray
LINAC: Linearbeschleuniger
MCS: Motor cortex stimulation
MVD: Mikrovaskuläre Dekompression
NCA: Neurochirurgische Akademie
PNS: Periphere Nervenstimulation
RF: Radiofrequenz
SCA: Arteria cerebelli superior
sTNFS: Subkutane Trigeminus Nerven- und Feldstimulation
tDCS: Transkranielle Direct-Current-Stimulation
TGN: Trigeminusneuralgie
THS: Tiefe Hirnstimulation
TKMS/TMS: Transkranielle Magnetstimulation
TOF-MRA: Time-of-Flight-MR-Angiographie

## References

[1] Bennetto L, Patel NK, Fuller G (2007) Trigeminal neuralgia and its management. BMJ 334:201–205 17255614 10.1136/bmj.39085.614792.BE PMC1782012

[2] Headache Classification Committee of the International Headache Society (IHS) (2018) The International Classification of Headache Disorders. Cephalalgia 38:1–211 (3rd edition) 10.1177/0333102417738202 29368949

[3] Ruscheweyh R, Gierthmühlen J, Hedderich DM et al (2024) Medikamentöse Therapie der Trigeminusneuralgie. Schmerz 38:283–292 38689064 10.1007/s00482-024-00810-4

[4] Mueller D, Obermann M, Yoon MS et al (2011) Prevalence of trigeminal neuralgia and persistent idiopathic facial pain: a population-based study. Cephalalgia 31:1542–1548 21960648 10.1177/0333102411424619

[5] Thomas KL, Vilensky JA (2014) The anatomy of vascular compression in trigeminal neuralgia. Clin Anat 27:89–93 23381734 10.1002/ca.22157

[6] Yamoto T, Nishibayashi H, Ogura M et al (2020) Three-dimensional morphology of the superior cerebellar artery running in trigeminal neuralgia. J Clin Neurosci 82:9–12 33317746 10.1016/j.jocn.2020.10.023

[7] Blitz AM, Northcutt B, Shin J et al (2018) Contrast-Enhanced CISS Imaging for Evaluation of Neurovascular Compression in Trigeminal Neuralgia: Improved Correlation with Symptoms and prediction of Surgical Outcomes. AJNR Am J Neuroradiol 39:1724–1732 30139749 10.3174/ajnr.A5743 PMC7655287

[8] Zhao ZF, Liu BB, Zhang JA et al (2023) Compression Degree of Trigeminal Nerve and Type of Conflicting Vessels Determine Short- and Long-Term Complete Pain Relief in Adult Patients with Primary Trigeminal Neuralgia After Microvascular Decompression: A Three-Year Retrospective Study of 200 Adult Patients with Primary Trigeminal Neuralgia. J Pain Res 16:4191–4207 38090024 10.2147/JPR.S429713 PMC10712259

[9] Antonini G, Di Pasquale A, Cruccu G et al (2014) Magnetic resonance imaging contribution for diagnosing symptomatic neurovascular contact in classical trigeminal neuralgia: a blinded case-control study and meta-analysis. Pain 155:1464–147110 24785270 10.1016/j.pain.2014.04.020

[10] Maarbjerg S, Wolfram F, Gozalov A et al (2015) Significance of neurovascular contact in classical trigeminal neuralgia. Brain 138:311–319 25541189 10.1093/brain/awu349

[11] DeSouza DD, Hodaie M, Davis KD (2016) Structural Magnetic Resonance Imaging. Can Identify Trigeminal System Abnormalities in Classical Trigeminal Neuralgia. Front Neuroanat 10(95) (3389/fnana.2016.00095) 10.3389/fnana.2016.00095 PMC5070392 27807409

[12] Zeng Q, Zhou Q, Liu Z, Li C, Ni S, Xue F (2013) Preoperative detection of the neurovascular relationship in trigeminal neuralgia using three-dimensional fast imaging employing steady-state acquisition (Fiesta) and magnetic resonance angiography (MRA). J Clin Neurosci 20:107–111 23098388 10.1016/j.jocn.2012.01.046

[13] AWMF Diagnostik und Therapie der Trigeminusneuralgie. https://www.awmf.org/service/awmf-aktuell/trigeminusneuralgie

[14] Dandy WE (1934) Concerning the case of trigeminal neuralgia. Am J Surg 24:447–455

[15] Gardner WJ (1962) Concerning the mechanism of trigeminal neuralgia and hemifacial spasm. J Neurosurg 19:947–958 13946557 10.3171/jns.1962.19.11.0947

[16] Jannetta PJ (1967) Arterial compression of the trigeminal nerve at the pons in patients with trigeminal neuralgia. J Neurosurg 26:159–162 10.3171/jns.1967.26.1part2.0159 6018932

[17] Di Carlo DT, Benedetto N, Perrini P et al (2023) Clinical outcome after microvascular decompression for trigeminal neuralgia: a systematic review and meta-analysis. Neurosurg Rev 46:8. 10.1007/s10143-022-01922-0 10.1007/s10143-022-01922-0 36481917

[18] Masuoka J, Matsushima T, Inoue K et al (2015) Outcome of microvascular decompression for trigeminal neuralgia treated with the stitched sling retraction technique. Neurosurg Rev 38:361–365 25663308 10.1007/s10143-015-0607-5

[19] Sindou M, Amrani F, Mertens P (1990) Microsurgical vascular decompression in trigeminal neuralgia. Comparison of 2 technical modalities and physiopathologic deductions. A study of 120 cases] [Article in French]. Neurochirurgie 36:16–25 2352588

[20] Sindou M, Leston JM, Decullier E et al (2008) Microvascular decompression for trigeminal neuralgia: the importance of a noncompressive technique—Kaplan-Meier analysis in a consecutive series of 330 patients. Neurosurgery 63(2):341–350 18981841 10.1227/01.NEU.0000327022.79171.D6

[21] Menna G, Rapisarda A, Izzo A et al (2022) Surgical and Clinical Outcomes of Microvascular Decompression: A Comparative Study between Young and Elderly Patients. Brain Sci 12:1216. 10.3390/brainsci12091216 36138952 10.3390/brainsci12091216 PMC9496765

[22] Liang C, Yang L, Zhang B et al (2023) 3D multimodal image fusion based on MRI in the preoperative evaluation of microvascular decompression: A meta-analysis. Exp Ther Medicine 25:171–178 10.3892/etm.2023.11870 PMC10061047 37006872

[23] Xie ME, Halbert-Eliott K, Nair SK et al (2024) Application of Sequential. Thresholding-Based Automated Reconstruction of the Trigeminal Nerve in Trigeminal Neuralgia. World Neurosurg 181:e567–e577 37890771 10.1016/j.wneu.2023.10.095 PMC11055639

[24] Sweet WH, Wepsic JG (1974) Controlled thermocoagulation of trigeminal ganglion and rootlets for differential destruction of pain fibers. 1. Trigeminal neuralgia. J Neurosurg 40:143–156 4587949 10.3171/jns.1974.40.2.0143

[25] Kanpolat Y, Savas A, Bekar A et al (2001) Percutaneous controlled radiofrequency trigeminal rhizotomy for the treatment of idiopathic trigeminal neuralgia: 25-year experience with 1600 patients. Neurosurgery 48:524–534 11270542 10.1097/00006123-200103000-00013

[26] Orhurhu V, Khan F, Quispe RC et al (2020) Use of Radiofrequency Ablation for the Management of Facial Pain: A Systematic Review. Pain Phys 23:E559–E580 33185371

[27] Texakalidis P, Xenos D, Tora MS et al (2019) Comparative safety and efficacy of percutaneous approaches for the treatment of trigeminal neuralgia: A systematic review and meta-analysis. Clin Neurol Neurosurg 182:112–122 31121470 10.1016/j.clineuro.2019.05.011

[28] Régis J, Tuleasca C, Resseguier N et al (2016) Long-term safety and efficacy of Gamma Knife surgery in classical trigeminal neuralgia: a 497-patient historical cohort study. J Neurosurg 124:1079–1086 26339857 10.3171/2015.2.JNS142144

[29] Pollock BE, Phuong LK, Foote RL et al (2001) High-dose trigeminal radiosurgery associated with increased risk of trigeminal nerve dysfunction. Neurosurgery 49:58–62 11440460 10.1097/00006123-200107000-00008

[30] Henson CF, Goldman HW, Robert H, Rosenwasser RH et al (2005) Glycerol rhizotomy versus gamma knife radiosurgery for the treatment of trigeminal neuralgia: an analysis of patients treated at one institution. Int J Radiat Oncol Biol Phys 63:82–90 16111575 10.1016/j.ijrobp.2005.01.033

[31] Alvarez-Pinzon AM, Wolf AL, Heather N et al (2017) Comparison of Percutaneous Retrogasserian Balloon Compression and Gamma Knife Radiosurgery for the Treatment of Trigeminal Neuralgia in Multiple Sclerosis. World Neurosurg 97:590–594 27756676 10.1016/j.wneu.2016.10.028

